# Elevated creatine kinase activity in primary hepatocellular carcinoma

**DOI:** 10.1186/1471-230X-5-9

**Published:** 2005-03-05

**Authors:** Georg Meffert, Frank N Gellerich, Raimund Margreiter, Markus Wyss

**Affiliations:** 1 Department of General and Transplant Surgery, University Hospital, Anichstrasse 35, A-6020 Innsbruck, Austria

## Abstract

**Background:**

Inconsistent findings have been reported on the occurrence and relevance of creatine kinase (CK) isoenzymes in mammalian liver cells. Part of this confusion might be due to induction of CK expression during metabolic and energetic stress.

**Methods:**

The specific activities and isoenzyme patterns of CK and adenylate kinase (AdK) were analysed in pathological liver tissue of patients undergoing orthotopic liver transplantation.

**Results:**

The brain-type, cytosolic BB-CK isoenzyme was detected in all liver specimens analysed. Conversely, CK activity was strongly increased and a mitochondrial CK (Mi-CK) isoenzyme was detected only in tissue samples of two primary hepatocellular carcinomas (HCCs).

**Conclusion:**

The findings do not support significant expression of CK in normal liver and most liver pathologies. Instead, many of the previous misconceptions in this field can be explained by interference from AdK isoenzymes. Moreover, the data suggest a possible interplay between p53 mutations, HCC, CK expression, and the growth-inhibitory effects of cyclocreatine in HCC. These results, if confirmed, could provide important hints at improved therapies and cures for HCC.

## Background

Creatine kinase (CK) isoenzymes catalyse the reversible transfer of the phosphate group of phosphocreatine (PCr) to ADP, to yield ATP and creatine (Cr). The CK/PCr/Cr system is present primarily in tissues with high and fluctuating energy demands such as brain, heart and skeletal muscle, and serves as a temporal and spatial "energy buffer" that helps to maintain a high intracellular phosphorylation potential in situations of increased metabolic demand (for reviews, see [1,2]).

In mammals, Cr can be taken up by the intestine from the food, or can be synthesized *de novo*. The liver is the main site of Cr production in the body (see [2]). After its synthesis, Cr is transported through the blood and is taken up by Cr-containing tissues via a specific Cr transporter. Whereas the importance of the liver in Cr biosynthesis is undisputed, some confusion still exists on the CK activity and PCr content in this organ. The majority of findings suggest no or minute levels of CK and PCr in liver tissue and, in particular, in hepatocytes (e.g., [3-6]). Other studies that used more sensitive experimental approaches provided evidence for low levels of PCr and CK, specifically localized in sinusoidal endothelial cells ([7-9]; see also [10]). Finally, in a few cases, more extreme findings were made: unusually high levels of CK activity were measured in liver tissue by Shatton et al. [11], Goullé et al. [12], and Wali & Makinde [13].

The majority of studies indicated that the low levels of CK activity in liver are due solely to the brain-type cytosolic CK (BB-CK) isoenzyme. On the other hand, besides BB-CK which was suggested to be present in endothelial and Kupffer cells, Vaubourdolle et al. [14] also provided evidence for the presence of the muscle-type cytosolic (MM-CK) isoenzyme in Ito cells, and for mitochondrial CK (Mi-CK) in hepatocytes. Similarly, Kanemitsu et al. [15] purified Mi-CK from normal human liver, which would imply significant amounts of this isoenzyme in liver tissue. Finally, increases in serum CK activity were frequently observed in cases of severe liver disease, with the most obvious source of CK being the pathological liver tissue itself [16-18].

In a number of studies reporting significant levels of CK activity in liver, interference by adenylate kinase (AdK) isoenzymes in the CK activity assays [19-21] is very likely (e.g., [13]), or can at least not be excluded, thus questioning the validity of these studies. Another possible cause for inconsistent findings might be compensatory up-regulation of CK expression in pathological liver tissue. Two lines of evidence that favour this hypothesis are: (i) partially hepatectomized rat liver was reported to show an increase in BB-CK activity (see [22]); and (ii) overexpression of CK isoenzymes in the liver of transgenic mice was shown to stabilize energy metabolism under low-oxygen stress and after a metabolic challenge [23,24], to accelerate regeneration of liver mass following major hepatectomy [10,25], and to increase endotoxin tolerance [5,26].

Because of these conflicting data, the goal of the present study was to analyse in detail the CK and AdK activities in pathological liver tissue of patients undergoing orthotopic liver transplantation.

## Methods

### Liver samples

The present project was approved by the ethics commission of the University of Innsbruck. In total, 25 liver samples were analysed. Twenty-three samples were obtained from 18 explanted organs of liver transplant recipients, one sample was obtained at autopsy (no. 1), and the last sample was from a normal rat liver. According to pathomorphological criteria, the 25 samples can be divided into 5 groups: (1) Nine samples of cirrhotic liver tissue (nos. 5, 7, 11, 13, 17–19, 23, 24: 4 due to hepatitis B or C virus infection, 3 due to primary or secondary biliary cirrhosis, 1 due to chronic alcohol abuse, and 1 due to vena hepatica occlusion); (2) six samples of neoplastic tissue (nos. 4, 9, 14, 15, 20, 21: 3 cholangiocellular carcinomas, 2 primary hepatocellular carcinomas, and 1 liver metastasis of a malignant melanoma); (3) three samples of necrotizing liver tissue due to acute or subacute organ rejection (nos. 2, 6, 22); (4) five samples of macroscopically normal liver parenchymal tissue (nos. 3, 8, 10, 16, 25) surrounding focal liver pathologies (i.e., 2 primary HCCs [samples 4 and 9]; metastasis of malignant melanoma [sample 15]; vena hepatica occlusion [sample 24]); (5) two samples originating from a normal rat liver (no. 12) and from a patient with steatosis hepatis (no. 1).

### Preparation of homogenate, cytosolic and mitochondrial fractions of human and rat liver

All steps were performed on ice or at 4°C. Approximately 5 g of liver tissue was homogenized in 45 ml buffer A (250 mM sucrose, 5 mM HEPES, 0.5 mM EGTA, pH 7.4). The homogenate was subjected to centrifugation for 5 min at 800 g. The pellet was discarded, and the supernatant centrifuged for 4 min at 5,100 g (centrifugation C2). The supernatant of C2 was further clarified by centrifugation for 12 min at 12,300 g, thus yielding the cytosolic fraction. The pellet of C2 was resuspended in 10 ml buffer A, followed by centrifugation for 2 min at 12,300 g (C3). After resuspension of the C3 pellet in 10 ml buffer A and centrifugation for a further 10 min at 12,300 g, the sediment was resuspended in 4 ml buffer A, thus yielding the mitochondrial fraction. One-ml aliquots of the different fractions were immediately frozen in liquid nitrogen and stored at -80°C until analysis.

### Measurements of CK and AdK activity

For CK and AdK activity measurements, the following assay medium was used: 110 mM imidazole, pH 6.7, 20.5 mM glucose, 11 mM Mg-acetate, 2.05 mM EDTA·Na_2_, 2.1 mM ADP, 2.1 mM NADP, 21 mM N-acetylcysteine, 9 U/ml of hexokinase, and 5.8 U/ml of glucose-6-phosphate dehydrogenase (both from Sigma). Enzymatic activity was measured at 25°C as an increase in NADPH absorbance at 340 nm. For AdK, three separate measurements were made for each sample in the same assay medium. For CK measurements, 5.1 mM AMP was added to the assay medium to inhibit AdK activity. For each sample, three measurements with 10.3 mM PCr and three measurements without PCr (blank measuring residual AdK activity after inhibition with AMP) were made, and the CK activity was calculated as the difference of the respective means. All values in this paper represent specific activities per mg of homogenate, cytosolic or mitochondrial protein. Protein amounts were measured according to the method of Bradford [27] with bovine serum albumin as standard.

### Cellulose polyacetate electrophoresis (CPE)

CPE was performed at room temperature for 90 min at a constant voltage of 150 V, but otherwise as described previously [28]. CK and AdK isoenzyme bands were visualized at 37°C with an overlay gel technique in a reaction protocol similar to the one described above for the measurement of enzymatic activity. NADPH was reacted with nitrobluetetrazolium in the presence of phenazine methosulfate to yield formazan. For visualization of CK bands, AMP was added to the overlay gel to inhibit AdK activity. Since AMP alone may not be sufficient to inhibit all AdK activity [21], two identical cellulose polyacetate strips were run; one was developed with PCr in the overlay gel, whereas for the other, PCr was omitted from the overlay gel (blank).

## Results

**Figure 1:**
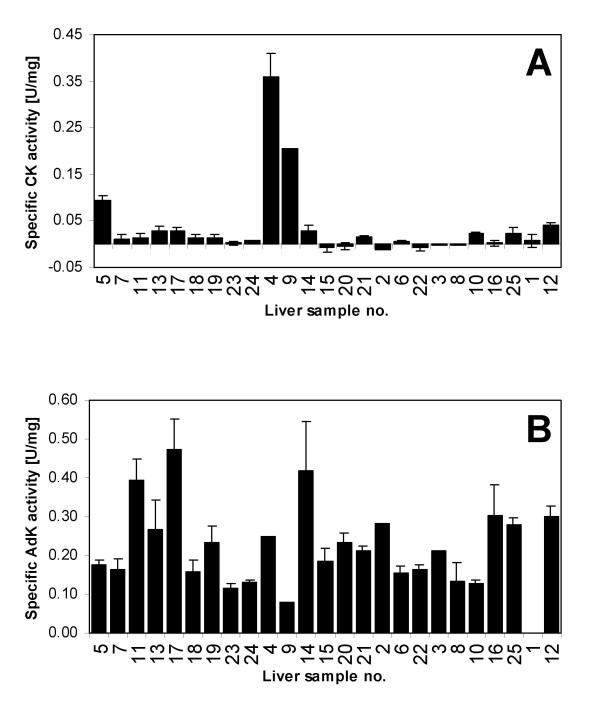
**CK and AdK activities in pathological and normal liver tissue. **Specific CK (A) and AdK activities (B) in total homogenate fractions were determined as described under "Methods". The liver samples were classified into five groups: group 1 – cirrhotic liver tissue (nos. 5, 7, 11, 13, 17–19, 23, 24); group 2 – neoplastic tissues (nos. 4, 9, 14, 15, 20, 21; 2 primary hepatocellular carcinomas [nos. 4 and 9]; 3 cholangiocellular carcinomas [nos. 14, 20 and 21]; 1 liver metastasis of a malignant melanoma [no. 15]); group 3 – samples of necrotizing liver tissue due to acute or subacute organ rejection (nos. 2, 6, 22); group 4 – samples of macroscopically normal liver parenchymal tissue (nos. 3, 8, 10, 16, 25) surrounding focal liver pathologies (2 primary HCCs [nos. 4 and 9]; metastasis of malignant melanoma [no. 15]; vena hepatica occlusion [no. 24]); group 5 – samples originating from a normal rat liver (no. 12) and from a patient with steatosis hepatis (no. 1). No AdK activity measurements are available for liver sample no. 1.

**Figure 2:**
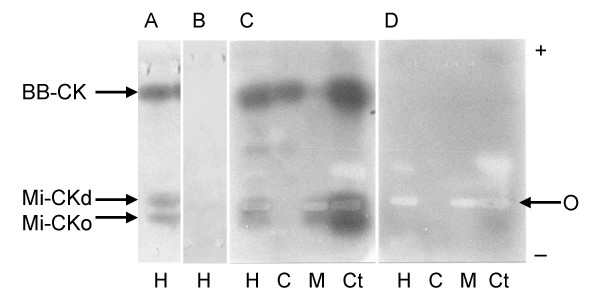
**CK isoenzymes in HCC. **Subcellular fractions of HCC liver samples no. 4 (A, B) and 9 (C, D) were analysed by cellulose polyacetate electrophoresis as described under "Methods". H = total homogenate; C = cytosolic fraction; M = mitochondrial fraction; Ct = control containing BB-CK and Mi-CK; Mi-CKd = dimeric Mi-CK; Mi-CKo = octameric Mi-CK; O = place of sample application. The cellulose polyacetate strips were exposed to an overlay gel containing PCr (A, C = CK activity staining), or lacking PCr (B, D = corresponding negative controls). Whereas Mi-CKd and Mi-CKo were well separated in (A), they were less so in (C).

CK and AdK activities were measured in total homogenate (Fig. 1), cytosolic and mitochondrial fractions (data not shown) obtained by differential centrifugation from 25 normal and pathological liver samples. Highest CK activities were observed in the two primary HCCs analysed (liver samples no. 4 and 9), with specific CK activities in the homogenate of 0.36 and 0.21 U·(mg protein)^-1^, respectively. In most other liver samples, the specific CK activities in the homogenate were below 0.05 U·(mg protein)^-1^. Whereas enzymatic activity measurements revealed low, but consistent CK activity in many of the cytosolic fractions, no CK activity was detected in the mitochondrial fractions, except for HCC sample no. 9 with a specific CK activity of approx. 0.1 U·(mg protein)^-1 ^(due to limited sample size, subcellular fractionation was not feasible for HCC sample no. 4). These findings were corroborated qualitatively by isoenzyme electrophoresis on cellulose polyacetate strips. Visualization of the different CK isoenzymes by an overlay gel technique revealed that the brain-type cytosolic BB-CK isoenzyme was present in all liver samples. Conversely, bands for the dimeric and octameric forms of Mi-CK were only observed in the two primary HCC samples (nos. 4 and 9; Fig. 2).

The CK/AdK activity ratio in the homogenate was 1.4 and 2.6 for the two primary HCCs (liver samples no. 4 and 9, respectively), 0.5 for liver sample no. 5 (secondary biliary cirrhosis), and < 0.2 for all other liver samples. Similar findings were made for the cytosolic and mitochondrial fractions, with CK/AdK activity ratios of < 0.3 for the cytosolic fraction and < 0.05 for the mitochondrial fraction. For HCC sample no. 9, however, these ratios were significantly higher: 4.8 (cytosolic fraction) and 0.33 (mitochondrial fraction).

## Discussion

CK is an enzyme still widely analysed in clinical diagnostics. Although a wealth of CK measurements have been reported in the scientific literature, there still exist inconsistency and incomplete knowledge on such an apparently simple question as the CK (isoenzyme) content of mammalian liver in both health and disease. In the present study, we detected the presence of BB-CK in all liver samples analysed by using CK activity measurements and cellulose polyacetate electrophoresis. However, in the normal and most pathological liver samples that we analysed, the specific CK activity was very low (< 0.05 U·[mg protein]^-1^), levels which are comparable with or lower than data reported for rat and human liver [29,30], but much lower than the specific CK activities in skeletal muscle, heart and brain (2–37 U·[mg protein]^-1^; [29,31-33]). We additionally observed that (i) the specific AdK activities in these samples were consistently higher than the specific CK activities (on average, > 10-fold), (ii) both activity measurements and cellulose polyacetate electrophoresis revealed similar specific AdK activities in the cytosolic and mitochondrial fractions (although from different AdK isoenzymes; data not shown), and (iii) mitochondrial respiration, in the presence of ATP, could be fully stimulated by AMP, but not by creatine (data not shown). This last observation favours the interpretation that in normal hepatocytes, CK isoenzymes are not expressed, and that the AdK isoenzyme system plays a function in high-energy phosphate buffering and transport, which is similar to the role of CK in brain, skeletal muscle and heart. Although histochemical data are missing, the results obtained here are most consistent with a localization of small amounts of BB-CK in sinusoidal endothelial cells [14].

Interestingly, we observed a strong induction of both BB-CK and Mi-CK expression in two samples of primary HCC. Despite CK/AdK activity ratios *in vitro *of 1.4–2.6, the specific CK activities were still relatively low (0.21–0.36 U·[mg protein]^-1^). Therefore, in the absence of histochemical data, it cannot be concluded with certainty whether the increased levels of CK are due to increased vascularization of the tumour (possibly associated with a higher proportion of CK-containing endothelial cells), or to induction of CK expression in the malignant cells.

Induction of CK expression has been observed previously in many types of tumours (see [2]) and may reflect an adaptation of the tumour tissue to the increased energetic demands. Evidence for induction of CK in liver tumours mostly comes from hepatoma cells grown in tissue culture [34,35] or, indirectly, from increased amounts of circulating BB-CK and Mi-CK in the blood of patients with liver tumours [36]. On the other hand, analysis of the tumour tissue itself, both by classical biochemical methods and by microarray technology, provided inconsistent results. Some authors reported induction of CK expression in liver tumours ([37-39]; and, in part, [40]; M. Sakamoto and S. H. Yim, personal communication), others repression [41], and still others observed no statistically significant differences between normal and malignant liver tissue [11,30]. This may be a reflection of the diverse clinicopathological and biological phenotypes of HCC, with different underlying molecular defects.

A key player in the picture might be the p53 tumour suppressor gene. Mutations in p53 are quite prevalent in HCC, especially in tumours with low cellular differentiation [42,43]. On the other hand, p53 was shown to control BB-CK expression: transrepression as observed for wild-type p53 is prevented by different mutations in the p53 gene [44]. Therefore, it is tempting to speculate that induction of BB-CK in HCCs is caused directly or indirectly by mutations in p53.

Expression of CK in HCC may have therapeutic implications, which is all the more important given (i) the limited responsiveness of HCC to currently available therapeutic approaches and, thus, (ii) the poor prognosis associated with this disease. Cr analogues (cyclocreatine and β-guanidinopropionic acid) and also Cr itself were previously shown to have antitumour activity, both in cell culture and in *in vivo *models ([45,46]; see also [2]). The responsiveness of tumour cells to growth inhibition by cyclocreatine seems to be correlated with their specific CK activity; cell lines with a specific CK activity of > 0.10 U·(mg protein)^-1 ^were generally sensitive to the drug. As for the liver, β-guanidinopropionic acid and creatine slowed the growth of AS30-D ascites tumour cells in culture (chemically induced rat hepatoma; [34]). Similarly, cyclocreatine revealed antitumour effects in a rat model of chemically induced hepatocarcinogenesis [47].

## Conclusion

The present findings shed light on some old enigmas and open up fascinating avenues for future research. Our findings do not support significant expression of CK in normal liver and most liver pathologies, but rather indicate that many of the previous misconceptions in this field can be explained by interference from AdK isoenzymes. On the other hand, given the need for improved understanding of the molecular pathogenesis of HCC, and for improved therapies and cures, the induction of CK expression in HCC described here calls for a more in-depth analysis of the interplay between p53 mutations, HCC, CK expression, and the growth-inhibitory effects of cyclocreatine in HCC.

## List of abbreviations

AdK, adenylate kinase; BB-CK, brain-type cytosolic CK isoenzyme; CK, creatine kinase; Cr, creatine; HCC, hepatocellular carcinoma; Mi-CK, mitochondrial CK; MM-CK, muscle-type cytosolic CK isoenzyme; PCr, phosphocreatine.

## Competing interests

The authors declare that they have no competing interests.

## Authors' contributions

GM and RM covered the medical part of this study. GM, FNG and MW performed the biochemical experiments. MW drafted the manuscript.

## Pre-publication history

The pre-publication history for this paper can be accessed here:
